# From free association to free parameters: machine-learning empowers artificial intelligence models to rate the affective-relational aspects of narratives like an expert psychologist

**DOI:** 10.3389/fdgth.2026.1762253

**Published:** 2026-08-27

**Authors:** Caleb J. Siefert, Barry Dauphin, Jenelle Slavin-Mulford, Sai Dileep Kumar Mukkamala, Venkat Akhila Reddy Tatipally, Areen Alsaid, Abdallah Chehade

**Affiliations:** 1 Department of Behavioral Sciences, University of Michigan-Dearborn, Dearborn, MI, United States; 2 Psychology Department, University of Detroit-Mercy, Detroit, MI, United States; 3 Department of Psychological Sciences, Augusta University, Augusta, GA, United States; 4 Department of Industrial and Manufacturing Systems Engineering, University of Michigan-Dearborn, Dearborn, MI, United States

**Keywords:** artificial intelligence, narrative assessment, narrative research, personality assessment, psychological assessment, social cognition and object relations scales—global rating method

## Abstract

**Background:**

Narrative assessment offers unique insight into psychological functioning but is resource-intensive, requiring extensive expert training and time. Recent work suggests Artificial Intelligence (AI), including moderately sized, adaptable large language models (LLMs), can master narrative assessment's complex, multi-step scoring rules. This study examined whether fine-tuning could enable AI raters to accurately assess narratives.

**Method:**

We fine-tuned five diverse LLMs (3–7 billion parameters) on two datasets, one for the SCORS-G Affective Quality of Representations (AFF) scale and one for Emotional Investment in Relationships (EIR). All narratives were rated by expert human raters. Each dataset was split into training and testing samples to evaluate AI-human agreement.

**Results:**

Fine-tuned models showed good to excellent reliability with human experts for both AFF and EIR. Ensemble ratings, averaged across all five models, yielded excellent single-rater and average-rater ICCs and low error rates, with 97% of AFF and 92% of EIR ratings falling within accepted discrepancy limits.

**Conclusion:**

Fine-tuning effectively adapted AI models into raters capable of reliably scoring complex psychological constructs from narrative. Ensemble-based AI raters can automate AFF and EIR ratings in research settings and, with further validation, may support clinical use via a human-in-the-loop approach. Fine-tuning adaptable AI models offers considerable potential to increase the feasibility and accessibility of sophisticated, multi-method assessment in research and clinical settings alike.

## 1 Introduction

Artificial intelligence (AI) and large language models (LLMs) have potential to rapidly transform key aspects of healthcare delivery, offering opportunities to enhance clinical decision-making, improve diagnostic accuracy, and streamline patient care processes (1, 2). Across health systems involving primary care, specialized medicine, and mental health, providers face mounting challenges from increased patient volumes, workforce shortages, demands for evidence-based practice, and navigating complex cases with considerable comorbidity. If AI technologies can provide viable support or tools to augment human expertise, this could increase efficiency, reduce the burden on clinicians, benefit patients, and even build useful databases (3, 4). By addressing longstanding challenges in service delivery and access (5, 6), AI assistants can extend the number of individuals assessed using highly sophisticated methods that rely on nuanced linguistic and thematic analysis. To realize these opportunities, research is needed to develop and assess the quality of AI tools. The present study explores whether AI raters, trained with a systematic, fine-tuning procedure, can interpret and rate narratives in a manner that aligns with ratings provided by advanced clinical psychologists.

Mental health assessment is a key component of comprehensive healthcare. It is also a resource-intensive and time-consuming aspects of practice (7). Traditional psychological assessments require extensive clinician training, significant time, and expertise that may not be readily available in all settings (8, 9). This creates barriers to timely and comprehensive evaluation, potentially delaying treatment initiation, and compromising patient outcomes (10). Currently, many health settings rely on ultra-brief screening tools, such as the PHQ-2 (11). Although these brief measures represent a step forward, their exclusive reliance on a single method (self-report) is a significant drawback. Multi-method assessment is considered a best practice in psychological assessment (12, 13). This approach involves the sophisticated integration of data from multiple sources, including performance-based tests, self-report measures, narrative analyses, and interviews. Such methods are time-consuming. They also require rigorous training and substantial domain expertise, which limits their use. AI tools have the potential to offset these logistical challenges.

AI has potential to render some sophisticated assessment methods, such as narrative assessment, which are more accessible for a wider range of clinical settings. Narrative assessment refers to systematic analysis of a patient's descriptions of events, stories told to pictures, autobiographical accounts, statements made during therapeutic discourse, and so on. It can offer insights into cognitive-emotional functioning, relationships, and psychological adaptation that can be difficult to capture through self-report (12–16). Despite research support for the clinical utility of narrative assessment (14, 17, 18), the training required to reach expert-level proficiency in such systems and time required to score them limit adoption in routine practice. Analyzing and rating narratives have historically required years of training and human expertise (14). Advances in modern LLMs' capacity for natural language processing create new possibilities for automating the nuanced text analyses. In healthcare, for example, LLMs show promise for extracting clinical information from records, supporting diagnostic decision-making, and enhancing patient communication (19–23), including NLP-based approaches to psychotherapy transcript analysis (24, 25). The present study builds on this emerging literature to examine whether smaller, adaptable LLM models can be fine-tuned to assess narratives for psychological constructs associated with well-being and health.

To assess AI's utility for use in assessment contexts, it is necessary to determine if AI raters can be developed and trained to draw conclusions that align with those from expert clinical psychologists. Narrative assessment systems, which contain highly complicated, multi-step rules for drawing inferences from narratives, may prove particularly challenging for AI in this respect. Thus, one way to address these issues is to see if LLMs can learn rating rules and rate narratives in ways that match ratings provided by human experts. One narrative rating system useful for this aim is the *Social Cognition and Object Relations Scales—Global Rating Method* (SCORS-G). The SCORS-G provides a framework for systematically evaluating psychological functioning through narrative analysis (14, 26, 27). Narratives are rated across eight dimensions of cognitive-emotional functioning. Scales assessing affective quality of representations (AFF) and emotional investment in relationships (EIR) are particularly central to mental health evaluation (26). SCORS-G ratings for EIR and AFF are associated with the degree of psychiatric distress (28), problems in social functioning (29), overall personality pathology (30), risk for hospitalization (31), and risk for self-harm (32–34). They are also useful for treatment planning. For example, SCORS-G ratings, and AFF and EIR specifically, at the start of treatment are associated with process variables later in treatment (35, 36), such as risk for premature termination (37) and treatment outcome (38). Changes in AFF and EIR have also been used to assess treatment progress over time (39). Studies have also examined the utility of SCORS-G assessments in primary care settings, with AFF and EIR both being associated with patient-physician interaction quality (40) and patient health care utilization (41). Evidence for associations between SCORS-G dimension and broad features of health also comes from non-clinical settings, where AFF and EIR have both been associated with indicators of health, well-being, and relational functioning (42, 43).

Despite empirical support, practical and logistical barriers often limit the routine use of narrative assessment scales, such as the SCORS-G, in healthcare and research settings. For example, narrative assessment systems have complex rating rules, requiring months to reach base proficiency and years to become expert. Providers may have neither the time nor access to obtain intensive training. The actual process of rating narratives is also resource-intensive, especially in research settings where multiple expert raters are often required. Further, reading narratives and providing ratings can be time consuming and incur considerable costs. The impacts of these challenges are felt in clinical settings and research contexts. The linguistic proficiency of LLMs may provide a potential solution to some of these challenges. For example, development of AI raters could increase the ease with which narrative-based assessments are conducted and quantified. A growing body of research suggests that modern LLMs may be indeed capable of making ratings in ways that align closely to human experts.

Research into the utility of LLMs for accurately rating psychological constructs has accelerated in the past several years. This rapidly emerging area of inquiry has shown considerable promise (44). Some studies have directed publicly available generative LLMs (e.g., ChatGPT) with prompts to achieve rating effectiveness. For example, Peters and Matz (45) used a zero-shot prompting approach with publicly available LLMs and found that they were able to rate Big Five personality traits derived from social media posts in ways that converged with respondent self-report. Simons and colleagues (46) showed that a zero-shot prompt-based approach, empowered GPT-3.5 to provide useful ratings of social attitudes, such as attitude certainty, importance, and moral conviction, by reviewing narrative statements from social media posts. Wright and colleagues (47) found that generative LLMs could assess personality traits from written narrative responses, with ratings converging with those provided by humans and real-world behavioral and mental health outcomes. Two studies (48, 49) have used expert-engineered prompts with publicly available LLMs (e.g., ChatGPT-4o; Claude-3.5-Sonnet) to direct them to rate narratives using the SCORS-G scales. Ratings from the AIs using these prompts showed strong agreement with those from expert psychologists with decades of experience. Like some prior studies (47, 50), agreement with experts was most robust when employing an ensemble approach that integrating ratings across multiple AI raters.

While prompt-engineering and zero-shot approaches have proven useful at directing publicly available LLMs to make assessments (45–49), this approach may have some limitations in healthcare settings. For example, this approach can incur considerable computational costs associated with rating large numbers of narratives. Further, narrative data would be visible to the companies that own the publicly available LLMs. This could pose issues for privacy and confidentiality. These approaches give developers little control over the AI raters they create, as publicly available LLMs' underlying weights can be altered by their owners at any moment and without warning. Such changes would necessitate reevaluation of the AI rater's reliability, accuracy, and validity. Thus, while research in this area is useful in many respects, it may have some limitations for deployment in healthcare settings.

An alternative to prompt engineering and zero-shot approaches involves fine-tuning adaptable AI models. This approach leverages transparent LLMs that can be trained to perform a specific task. After they have been trained, these models can run on local servers or local computers, increasing capacity for privacy. Further, these models are non-proprietary. Once they are fine-tuned, they cannot be arbitrarily changed by third parties. As such, they are more stable and easy to deploy. Despite these advantages over prompt-engineering and zero-shot approaches, fine-tuning can have some risks. First, adaptable AI models trained by healthcare professionals are often much smaller, in terms of the number of parameters they contain, relative to publicly available, proprietary counterparts (which can possess 100s of billions to trillions of parameters). While a simplification in some ways, an LLM's parameter count is correlated with its linguistic and reasoning capabilities. This could cause some to question whether these LLMs can rate nuanced narrative material (e.g., social media posts; clinical interviews; life history interviews) using highly complex rating systems regularly employed in healthcare and psychology. Several studies, however, suggest that this is possible. Oltmanns and colleagues (51) fine-tuned a model to assess Big Five personality traits using data from life narrative interviews and found that the models ratings were associated with self-report ratings provided by respondents. Galatzer-Levy and colleagues (52) used an LLM that had pre-tuned for medical language and trained it to effectively detect depression symptoms from clinical interview transcripts. A notable example of how effective fine-tuning can be effective for highly complex rating system and nuanced data was provided by Na and colleagues (50) who had engineering teams compete to fine-tune LLMs to identify and rate defense from conversational text using the Defense Mechanism Rating Scales (DMRS). Several teams were able to models that provided ratings aligning with those from human experts. One prior study directly explored if fine-tuning GPT-2, a relatively small LLM, could empower it to rate narratives using the SCORS-G's AFF scale (53). The results were promising. Following training, the model showed strong agreement with ratings provided by human experts and 84.7% of the model's ratings were within acceptable limits of expert scores. Although this study was a preliminary proof-of-concept with limitations (e.g., only trained one AI rater; small training sample sizes), the findings suggest that fine-tuning could empower even relatively small LLMs to rate SCORS-G scales effectively.

The present study builds on earlier work in important ways. It uses a much larger set of training and testing narratives relative to prior work with AFF (53). Instead of relying on a single model, we fine-tuned five AI raters to create an ensemble. We also used more recently developed adaptable LMMs, each being an “instruct” model. Rather than focusing on one SCORS-G dimension, we explored two: Affective Quality of Representations (AFF) and Emotional Investment in Relationships (EIR). Table 1 contains descriptions of these dimensions. Each dimension has a fully independent set of rating rules. We selected the AFF and EIR scales for a few reasons. First, both dimensions load onto the same affective-relational factor (54), enabling direct comparison of performance across two conceptually related but distinct constructs. Second, AFF and EIR are among the most clinically significant SCORS-G dimensions, producing more consistent associations with psychiatric distress, factors associated with risk, treatment processes variables (e.g., early drop out; therapeutic alliance), and health outcomes (28–43). Third, these scales are easier for humans to achieve proficiency, and we assumed the same might be true for AI. Thus, we wanted to see examine fine-tuning effectiveness for these “relatively easier” scales prior to extending the approach to the remaining SCORS-G dimensions which are more complex. Our overall goal was to see if fine-tuning procedures could empower AI raters to generate ratings that align with those from human experts.

**Table 1:** Descriptions of the SCORS-G Scales.

Scale	Core Concept Assessed	High-End Characteristics (High Scores)	Low-End Characteristics (Low Scores)
Affective Quality of Representations (AFF)	Emotional tone for how an individual frames their thinking about relationships.	Relational interactions are characterized as benevolent, caring, supportive, warm, and safe.	Relational interactions are depicted to be malevolent, hostile, dangerous, exploitative, or painful.
Emotional Investment in Relationships (EIR)	Depth and quality of Relationship commitment, and nature of interaction.	Characters show investment in others, value intimacy, are deeply connected, and interact warmly.	Relationships are depicted as superficial, primarily need-gratifying, or devoid of connection and closeness.

## 2 Method

### 2.1 The social cognitions and object relations scales—global rating method

The SCORS-G is an empirically validated system used by clinicians and researchers to assess core dimensions of personality functioning (14, 26, 27). It contains eight dimensions that can be used to rate narrative material, such as stories, autobiographical memories, relational episodes, or psychotherapy transcripts. All SCORS-G dimensions are rated on a one-to-seven scale. While specifics differ across dimensions, lower scores are indicative of poor or immature functioning, and higher scores suggest more adaptive or mature functional capacity. In addition to the eight dimensions, a global score can also be calculated. Factor analytic work with the SCORS-G suggests it is composed of a cognitive factor, a self-factor, and an affective-relational factor (54). This study focuses on the two effective-relational factor dimensions: Affective Quality of Representations (AFF) and Emotional Investment in Relationships (EIR). Brief descriptions of these dimensions are included in Table 1. For a more detailed description, see (14).

### 2.2 Artificial intelligence models for fine tuning

To develop AI raters, we selected five instruction-tuned LLMs: OpenChat [OpenChat/OpenChat-3.5-1210 (55), Google's Gemma [Google/gemma-7b-it (56), Mistral's Mistral-7B [Mistralai/Mistral-7B-Instruct-v0.2 (57), Alibaba Cloud's Qwen [Qwen/Qwen2.5-7B-Instruct-1M (58), and Meta's Llama-3.2 [Meta-llama/Llama-3.2-3B-Instruct (59). Each model is a cutting-edge, open-weight LLM in the roughly 3-to-7 billion parameter range. Collectively they contain diverse features, distinct architectural nuances, and were pre-trained under varied training philosophies. For example, Mistral-7B leverages Sliding Window Attention (SWA) and Grouped-Query Attention (GQA). Qwen2.5 has a very large context window. Gemma-7B was derived from the same highly rigorous pretraining approach used to create Google's Gemini models. OpenChat is a fine-tuned Mistral model that has undergone advanced, open-source fine-tuning which can provide a better starting point for specialized fine-tuning. Given that we anticipated using an ensemble approach, in which ratings from each individual AI rater is averaged into an overall rating, diversity in features, pretraining, and architecture was viewed as desirable.

### 2.3 Dataset description

We collected narratives from three different sources referred to as Sample A, B, and C (described below). A total of 1,966 narratives were collected with AFF ratings. Of these, we allocated 1,572 (80%) to our AFF training sample and 394 (20%) to our AF*F* testing sample. A total of 1,986 narratives were collected with EIR ratings. Of these, 1,587 (80%) used for training and 399 (20%) used for testing. When assigning narratives to the training and testing samples, narratives were randomized with efforts to have each sample contribute roughly 80% of its narratives to the training sample and roughly 20% of the narratives to the testing sample, while also distributing a roughly equal number of ratings along the 1–7 scale.

**Figure 1:**
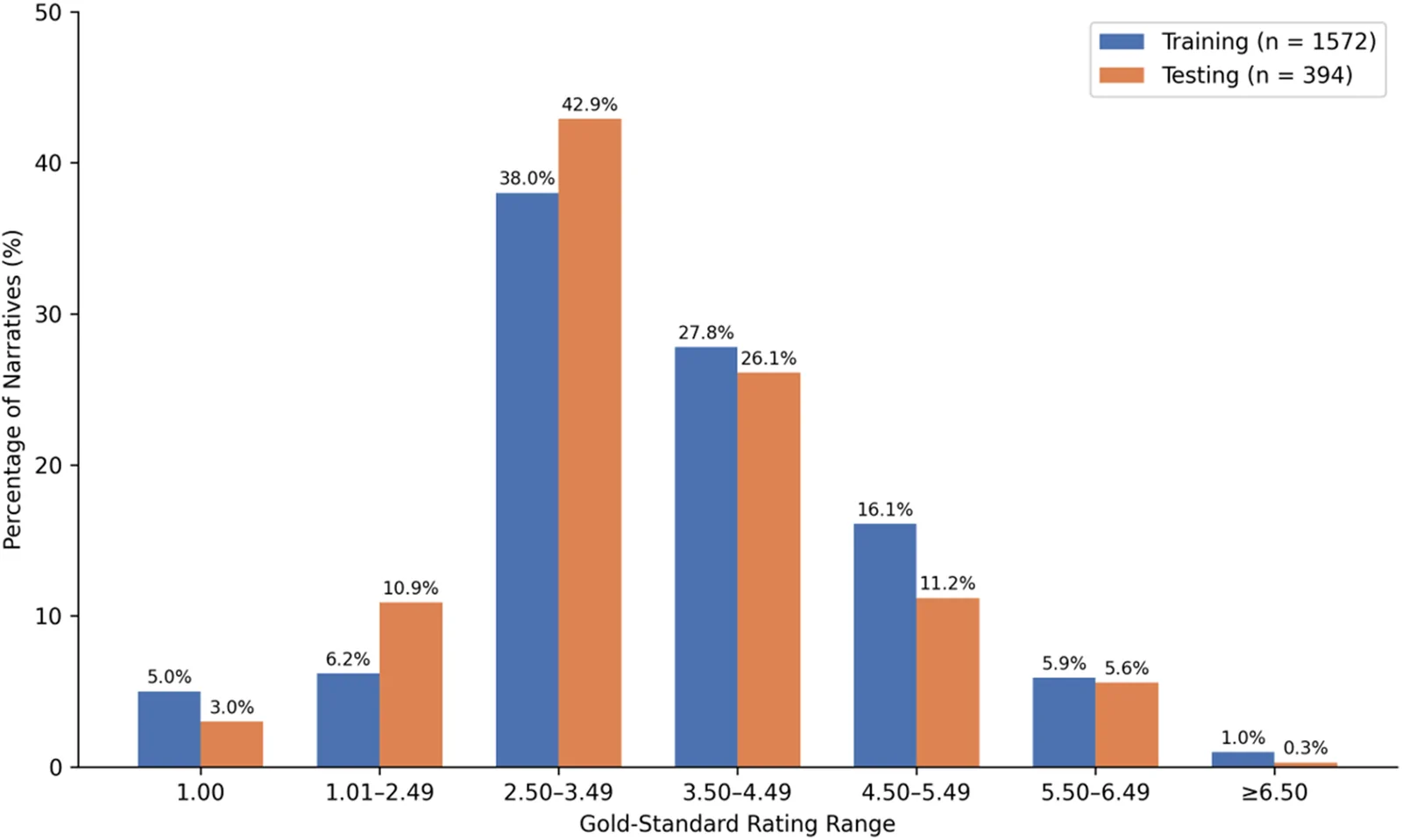
Distribution of affective quality of representation (AFF) ratings across the testing and training samples.

**Figure 2:**
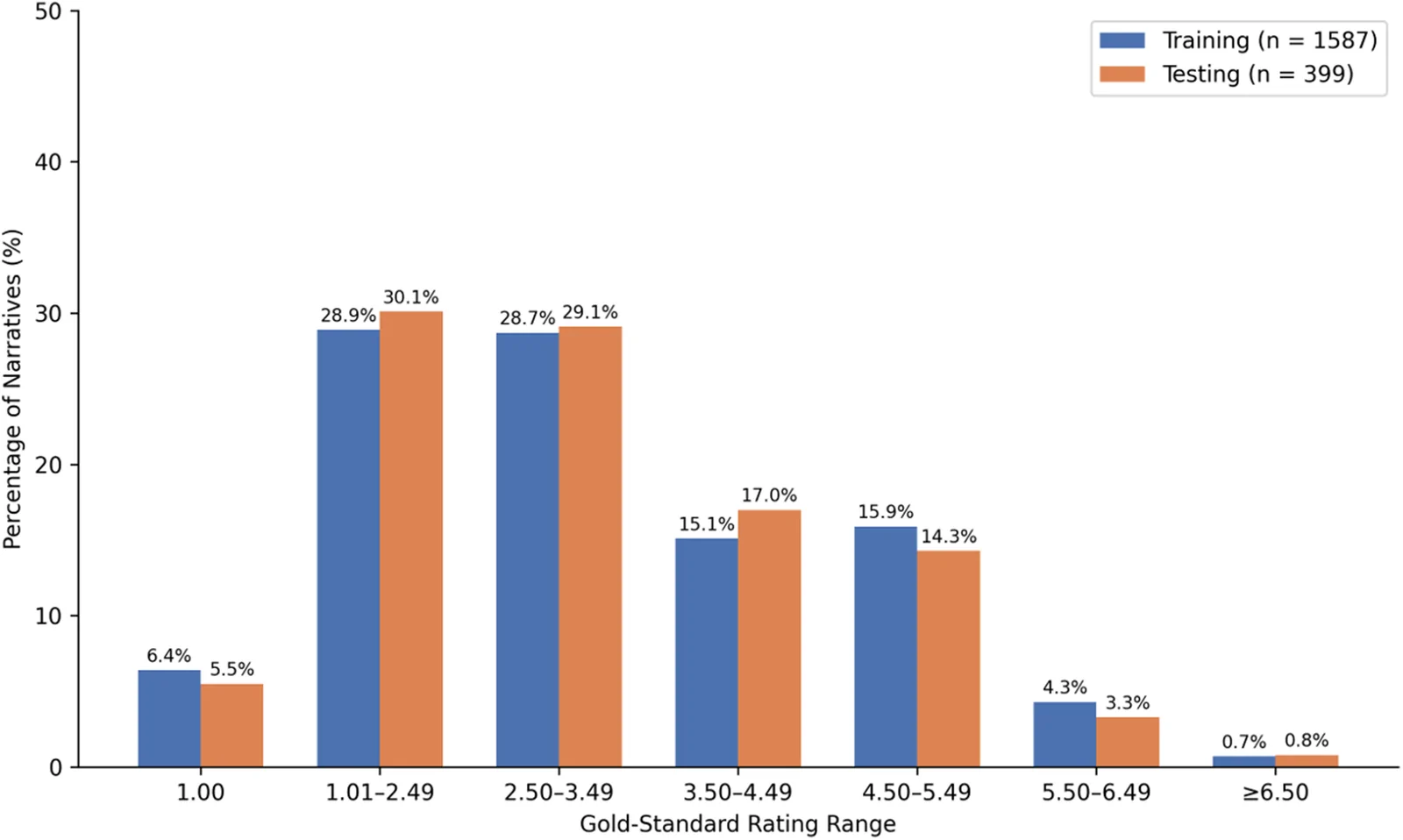
Distribution of emotional investment in relationships (EIR) ratings across the testing and training samples.

Across the three samples, all narratives were rated by different sets of expert raters. Each expert rater had completed training in the SCORS-G, demonstrated proficiency, and served as expert raters for several other studies. Although human raters assign integer ratings from 1 to 7, we used the average of all raters as our criterion rating for both training and testing purposes. As such, the criterion rating could contain a decimal value (e.g., 3.5; 4.5). Thus, we used score ranges, treating a rating of 1 as one level, ratings of 1.01–2.49 as the next level, ratings of 2.5–3.49 as the next level, ratings of 3.5–4.49 as the next level, ratings of 4.5–5.49 as the next level, ratings of 5.5–6.49 as the next level, and ratings of 6.5–7.0 as the top level. Distributions of rating ranges within the training and testing samples for AFF and EIR can be seen in Figures 1, 2, respectively.

Sample A were narratives from SCORS-G training materials (14, 26). These narratives are used to train and test human rater proficiency. Sample A provided 195 narratives rated for AFF, and we allocated 155 (79%) of these to the training dataset and 40 (21%) to the testing dataset. Of these, 120 narratives were stories told to Thematic Apperception Test cards (60) and 75 were early memories. Sample A also contributed 169 narratives with EIR ratings. Of these, 139 were allocated to the training sample (82%) and 30 to the testing sample (18%). These narratives included 105 stories told to TAT cards and 64 early memories. All narratives for Sample A were rated by human psychologists who helped develop the SCORS-G training materials and rating system (26).

Sample B contained narratives collected as part of an ongoing study focusing on young adults (61, 62). A total of 637 narratives were rated for AFF. We allocated 504 (79%) to the training sample and 133 (21%) to the testing sample. For EIR, 693 narratives with EIR codes were gathered from this sample, and 567 (82%) were assigned to the training sample and 126 (18%) were assigned to the testing sample. All narratives for Sample B were generated in response to TAT cards. For Sample B, two expert raters who had achieved excellent reliability and proficiency rated all narratives, and showed strong evidence of interrater reliability (61, 62).

Sample C contained narratives from a separate study at a different institution that also focused on young adults (54). All narratives were also generated in response to six TAT cards and one picture card (depicting two women in lab coats working in a laboratory (63). A total of 1,134 narratives were rated for AFF, with 913 (81%) assigned to the training sample and 221 (19%) assigned to the testing sample. Sample C contributed 1,124 EIR narratives, with 881 (78%) assigned to the training sample and 243 (22%) allocated to the testing sample. For Sample C, two clinical psychologists, each with over 15 years of experience using the SCORS-G, rated all narratives and demonstrated good to excellent interrater agreement (54).

Across all samples, scores were averaged across raters to create a criterion rating from human experts. These criterion ratings served as “gold-standard” ratings for training and testing purposes. Inclusion of narratives rated by multiple expert groups (vs. including only narratives rated by a single expert set) is advantageous in that it increases the likelihood that AI rater proficiency would generalize to human experts at large, rather than a specific group of raters.

### 2.4 Fine-tuning procedure

Transfer learning was adopted to introduce knowledge to shift LLMs from a general-purpose model to a task-specific model. Although the training data differed, the same fine-tuning approach was used to train AI raters for EIR and AFF; each model was subjected to an identical fine-tuning protocol to isolate performance differences attributable to the base model's architecture and pre-training. All fine-tuning was conducted in Google Colab Pro + on a single NVIDIA A100 GPU (High-RAM configuration). The software stack was built on PyTorch and the Hugging Face ecosystem, utilizing Parameter-Efficient Fine-Tuning (PEFT), datasets, accelerate, and bitsandbytes. PEFT keeps the majority of a model's parameters frozen (i.e., unchanged) while inserting tiny, trainable Low-Rank Adaptation (LoRA) adapters (1%) into its transformer architecture, allowing a model to be customized in a manner expedient for consumer-grade GPUs. Each model/dimension combination completed fine-tuning in approximately 30–45 min. The datasets library was used to load and preprocess the narrative-rating training data, while accelerate handled hardware orchestration during training. To make fine-tuning computationally feasible on a single GPU, each model was loaded with 4-bit NormalFloat4 (NF4) quantization via bitsandbytes, with computations performed in bfloat16 precision to improve efficiency. The model's tokenizer was configured to use the end-of-sequence token as the padding token to handle variable-length inputs. This method keeps the original model weights frozen while introducing small, trainable adapter matrices into its transformer architecture. By leveraging these library tools, the task of customizing a massive AI model with billions of parameters becomes achievable.

We employed a PEFT using LoRA to adapt base models. The LoRA configuration was explicitly defined with a rank of 16 and a scaling factor of 32. The trainable adapter layers were applied to all linear projection layers within the model's attention blocks: the query, key, value, and output projections, as well as the feed-forward network's gate, up projections, and down projections. A dropout rate of 5% was applied to the LoRA layers to prevent overfitting. This configuration notably limits the number of trainable parameters (less than 1% of the total model size), which drastically reduces memory and computational requirements. Regarding hyperparameters, each model was fine-tuned for three epochs. We used an AdamW optimizer with a linear learning rate scheduler, a learning rate of 1e-4, and a warmup ratio of 0.1. To manage GPU memory, we set the per-device training batch size to 1 and used gradient accumulation with two steps, producing an effective batch size of 2. The final fine-tuned LoRA adapter weights were saved and subsequently uploaded to the Hugging Face Hub for deployment.

The hyperparameters described above were not chosen arbitrarily; they reflect established practices for LoRA and QLoRA fine-tuning (64, 65). Specifically, rank 16 with alpha 32 is a commonly used LoRA configuration that balances representational expressivity with parameter efficiency. A dropout rate of 5% provides light regularization. A learning rate of 1e-4 with a linear schedule and 10% warmup is a standard AdamW configuration for instruction fine-tuning. Three epochs provide sufficient training exposure while guarding against overfitting, and gradient accumulation of 2 steps with a per-device batch size of 1 maintains stable gradient estimates within GPU memory constraints. In informal preliminary testing, performance appeared robust to reasonable variations in these settings, though this was not systematically evaluated. The decision to apply an identical protocol across all five models was deliberate, enabling a controlled architecture comparison in which performance differences can be attributed to the base model rather than to differences in fine-tuning configuration.

The design incorporates multiple safeguards against overfitting. First, LoRA at rank 16 updates only approximately 1.1% of parameters (≈41.9M of 3.8B), limiting the model's capacity to memorize training examples. Second, we applied 5% LoRA dropout, used a small effective batch size (2), and implemented a linear learning rate schedule with 10% warmup. We also trained for a fixed, short schedule of only 3 epochs rather than dynamically determining a stopping point through validation-loss curve monitoring or early stopping; we acknowledge this as a limitation of the current design. Together, these choices represent a deliberately conservative fine-tuning protocol. Critically, generalization performance was evaluated on a held-out test set never seen during training.

Separate CSV files were created for the training and testing data for AFF and EIR. Training data was prepared and loaded in from these CSV files. A structured prompting strategy was implemented to format the data for causal language modeling. Each training example was converted into a single string following a template similar to Chat Markup Language (ChatML) template. This template prepended a brief instructional prompt (the AFF prompt and EIR prompt can be viewed in Supplemental Material 1), which included scoring rules for each level of the target dimension (e.g., EIR). Each prompt served to orient the model to the task and provided a basic description of each rating level. Thus, prompts were not merely a formatting template; each provided an abbreviated rubric for its respective scale. The model was fine-tuned via causal language modeling to generate the numeric rating from this combined input, with the identical prompt used at both training and inference, so the model learned the mapping from (rubric + narrative) → rating. Thus, the prompt supplied fixed context that defines the rating criteria, and the fine-tuning taught the model to apply criteria to novel narratives. This approach has been shown to enhance reliability (66). This entire formatted string was tokenized. The model was trained to predict the next token in the sequence, thereby learning to generate the numeric rating, based on the provided narrative and instructional prompt.

### 2.5 Model testing procedures

To test AI rater performance following fine-tuning completion, we compared ratings from AI raters to criterion ratings from human experts by calculating intraclass correlation coefficients (ICC). ICCs were calculated with two-way random effects ICC models set for absolute agreement. We calculated inter-rater reliability estimates using both the single-rater [ICC (2,1)] formula and average rater (ICC, 2, k) formula. These quantify the level of agreement between AI raters and criterion ratings provided by human experts in the testing sample. We calculated ICCs for each AI rater (e.g., ICC for agreement between OpenChat rating and the criterion rating from human experts) and at the level of the AI ensemble rating. Note that we first averaged ratings across AI raters to generate create a single ensemble rating. We then calculated ICCs using the ensemble rating and criterion ratings from the human experts. Thus, even the ensemble rating contained only two raters (Ensemble and Human Experts).

We calculated both single-rater and average-rater ICCs given that AI raters can be used in different ways. Single-rater ICC formulas estimate reliability if only one rater, from a pool of raters, will assign ratings. Single-rater ICC estimates are useful for assessing AI's potential to fully automate ratings (i.e., no human involvement in rating). By contrast, the average-rater ICC formulas estimate reliability under the assumption that all raters in a pool will provide ratings, and these will be averaged to produce a final score. Average-rater estimates are useful for characterizing AI's potential in a *human-in-the-loop approach* where both a human expert and the AI ensemble independently rate a narrative and average scores to produce a final rating. While there is no consensus regarding the amount of rater agreement necessary to establish rater proficiency, guidelines have been suggested. For example, Cicchetti (67) characterizes ICC ranges as follows: < 0.40 = Poor, 0.40–.59 = Fair,.60–.74 = Good, and ≥ .75 = Excellent. Koo and Li (68) suggest more conservative ranges: <.50 = Poor,.50–.75 = Moderate,.75–.90 = Good, and >.90 = Excellent. The minimum level of acceptable agreement for research contexts for the SCORS-G is.60, with higher ICCs (i.e., ≥ .75) being seen as more desirable and common.

Beyond ICCs, we also examined both absolute discrepancies and signed discrepancies. Absolute discrepancy refers to the difference between the AI's rating and the criterion rating. For example, if the criterion rating from human experts is five and the AI ensemble rating is six, the absolute discrepancy would be one. Absolute discrepancy is considered in evaluating human proficiency and readiness for providing ratings (14, 26). Discrepancies of one or less are within accepted tolerance limits. Ideally, ninety percent of narratives or more should have a discrepancy of one or less. Absolute discrepancies of two or more are considered critical errors. Ideally, these should be quite rare, occurring at a rate of less than a 5%. While not as problematic as critical errors, rates of mild to moderate deviations should also be low. Thus, for each scale, we report the proportion of ratings within each of the following ranges: no discrepancy (discrepancies of 0.00); within tolerance limits (discrepancies of 0.01–1.00); mild deviation (discrepancies of 1.01–1.50); moderate deviation (discrepancies of 1.51–1.99); and critical errors (discrepancies ≥ 2.00).

Signed discrepancies were calculated by taking the expert rating and subtracting the predicted rating from the AI rater or ensemble. Thus, in our study, a positive average signed discrepancy would indicate tendencies to underrate (i.e., rate lower than human experts) and negative average signed discrepancy would indicate tendencies to overrate (i.e., rate higher than human experts). To evaluate signed discrepancy, we also conducted one-sample *t*-tests (set to consider if the difference obtained is significantly different from zero) to determine if average differences occurred by chance and considered Cohen's *d* to characterize the magnitude of effects using the thresholds provided by Sawilowsky (69): very small (*d* < 0.20.), small (*d* ≥ 0.20), medium (*d* ≥ 0.50), large (*d* ≥ 0.80), very large (*d* ≥ 1.20), and huge (*d* ≥ 2.00).

### 2.6 Evaluating the impacts of fine-tuning

To assess the extent to which the fine-tuning training procedure improved LLM performance, we examined performance for models prior to fine-tuning and compared these with performance after fine-tuning. To assess performance prior to fine tuning, models were given the same prepended prompts used for fine-tuning (see Supplemental Material 1) and instructed to rate narratives in the testing sample. If fine-tuning is improving performance, after fine-tuning ICCs should be larger and absolute discrepancies lower. We also anticipated that the fine-tuning would yield greater agreement among the five AI raters within each respective ensemble. Thus, we compared within-ensemble average-rater ICCs prior to and after fine-tuning.

## 3 Results

### 3.1 AI rater reliability with criterion ratings from human experts

The left portion of Table 2 reports single-rater ICCs, with 95% confidence intervals, for all AI raters and ensemble ratings. For AFF, single-rater reliability estimates (ICCs 2,1) ranged from.84 to.89, indicating good reliability for all five models. The ensemble rating ICC (2,1) was.91 indicating excellent reliability with the human experts. Single-rater ICCs (2,1) for AI raters assessing EIR ranged from.79 to.82, with an ensemble ICC (2,1) of.84. The right side of Table 2 reports average-rater ICCs for individual AI raters and the ensemble rating. For the AFF dimension, ICCs (2,2) for individual AI raters ranged from.91 to.94, and the ensemble rating reached an exceptional ICC (2,2) of.95. Strong reliability was also observed for EIR, where ICCs (2,2) ranged from.88 to.90, and the ensemble achieved an ICC (2,2) of.92.

**Table 2:** Single-Rater and average-rater intraclass correlation coefficients and 95% confidence intervals for post-fine-tuning LLMs and ensembles *.*

	Single-Rater Intraclass Correlation Coefficients (ICC, 2,1)				Average-Rater Intraclass correlation coefficients (ICCs, 2,2)			
AI Rater	AFF ICC	95% CI	EIR ICC	95% CI	AFF ICC	95% CI	EIR ICC	95% CI
OpenChat	.89	.87–.91	.82	.79–.86	.94	.93–.95	.90	.88–.92
Mistral	.89	.86–.91	.82	.78–.85	.94	.93–.95	.90	.88–.92
Gemma	.87	.84–.89	.81	.77–.84	.93	.91–.94	.89	.87–.91
Qwen	.86	.83–.88	.80	.74–.84	.92	.91–.94	.89	.85–.92
Llama	.84	.80–.86	.79	.74–.82	.91	.89–.93	.88	.85–.90
**Ensemble**	.**91**	**.89**–**.92**	.**84**	**.81**–**.87**	.**95**	**.94**–**.96**	.**92**	**.89**–**.93**

AFF, Affective Quality of Representations Dimension; EIR, Emotional Investment in Relationships Dimension; ICC, Intraclass Correlation Coefficient; 95% CI, 95% Confidence Interval for ICC.

### 3.2 Absolute and signed discrepancy

The left side of Table 3 reports the mean absolute discrepancies, standard deviation, and 95% confidence interval [based on standard error of measurement (SEM)] for absolute discrepancies between AI raters and criterion ratings provided by human experts in the testing samples. Findings indicate a high level of performance across all AI raters and the overall ensemble rating. For AFF, mean discrepancies ranged from.27 to.37, with the ensemble rating having a mean discrepancy of.29 (*SD* = .37). For EIR, mean discrepancies were slightly higher than those for AFF ranging from.47 to.51, with a mean discrepancy for the ensemble score of.45 (*SD* = .47). The right side of Table 3 reports the percentages of absolute discrepancy scores within various ranges. Both ensemble scores performed admirably with > 90% of ratings falling within the tolerance range. For AFF, 97% of ensemble ratings were within tolerance. Critical errors were nonexistent. For EIR, 92% of ensemble ratings were within tolerance limits. Only 1% of ratings were critical errors. For both scales, the error margin was small and composed of mild and moderate deviations.

**Table 3:** Distribution of absolute discrepancies and percentage of ratings with absolute discrepancy ranges.

AFF	Mean	SD	95% CI	Total % Within Limits	No Discrepancy	Within Limits	Mild Deviation	Moderate Deviation	Critical Error
OpenChat	.27	.43	.23–.31	97%	64%	33%	2%	0%	1%
Mistral	.29	.44	.25–.33	97%	64%	33%	2%	0%	1%
Gemma	.32	.47	.27–.37	96%	61%	34%	3%	0%	2%
Qwen	.34	.48	.29–.39	95%	59%	36%	3%	0%	2%
Llama	.37	.50	.32–.42	95%	58%	37%	4%	0%	2%
**Ensemble**	.**29**	.**37**	**.25**–**.33**	**97%**	**44%**	**53%**	**2%**	**1%**	**<** **1%**

EIR	Mean	SD	95% CI	Total % Within Limits	No Discrepancy Limits	Within Limits	Mild Deviation	Moderate Deviation	Critical Error
OpenChat	.48	.53	.43–.53	95%	45%	49%	3%	0%	3%
Mistral	.47	.55	.42–.54	96%	46%	50%	1%	0%	3%
Gemma	.48	.57	.42–.54	93%	48%	44%	3%	0%	4%
Qwen	.50	.57	.44–.54	92%	47%	45%	4%	0%	4%
Llama	.51	.58	.45–.57	92%	46%	46%	4%	0%	4%
**Ensemble**	.**45**	.**47**	**.40–50**	**92%**	**29%**	**63%**	**5%**	**2%**	**1%**

Mean, Average absolute discrepancy across all ratings in the testing sample; SD, standard deviation of absolute discrepancy ratings across all ratings in the testing sample; 95% CI, 95% confidence interval based on the standard error of measurement SEM for all absolute discrepancies for ratings in the testing sample; Total % Within Limits, percentage of ratings in the testing sample with an absolute deviation from zero to one; No discrepancy, percentage of testing sample ratings with an absolute discrepancy of zero; Within Tolerance, percentage of testing sample ratings with an absolute discrepancy between.01 and one; Mild Deviation, percentage of testing sample ratings with an absolute discrepancy of 1.01 to 1.50; Moderate Discrepancy, percentage of testing sample ratings with an absolute discrepancy absolute discrepancy of 1.51 to 1.99; Critical Error, percentage of testing sample ratings with an absolute discrepancy of two or more.

To assess systematic directional bias in how the ensembles (and specific AI raters) assign ratings, we calculated signed discrepancy by subtracting the ensemble's rating from the criterion rating. We did this separately for EIR and AFF. Results are shown in Table 4. Overall, findings suggest tendencies to for AI raters to provide ratings that are slightly below those provided by human experts. At the ensemble level, the mean signed discrepancy for AFF was small but significantly greater than zero [*M* = .06, *SD* = .46, 95% CI (.02,.11)], *t*(393) = 2.76, *p* < .01) but the magnitude of effect was small (*d* = .14). A similar, slightly larger pattern emerged for EIR, where the ensemble's mean signed discrepancy of.15 (*SD* = .64, 95% CI [.08,.21]) also differed significantly from zero (*t*(398) = 4.60, *p* < .001). Again, effect magnitude was small (*d* = .23). This pattern was consistent across individual AI raters as well: mean signed discrepancies ranged from -.01 to.12 for AFF and from.09 to.22 for EIR, suggesting the modest tendency toward underrating relative to human experts was a shared characteristic across models rather than one driven by a single rater.

**Table 4:** Descriptive statistics and tests of mean signed discrepancy by rater.

AFF	Mean	SD	95% CI	OS *t*	*p*	*d*
OpenChat	-.01	.51	-.06–.04	-.39	.70	-.02
Mistral	.07	.52	.02–.13	2.81	.01	.14
Gemma	.12	.56	.07–.18	4.28	<.001	.22
Qwen	.05	.59	-.01–.11	1.72	.087	.09
Llama	.08	.61	.02–.14	2.72	.007	.14
**Ensemble**	.**06**	.**46**	**.02**–**.11**	**2**.**76**	**.006**	.**14**

EIR	Mean	SD	95% CI	OS *t*	*p*	*d*
OpenChat	.14	.71	.07–.21	3.94	<.001	.20
Mistral	.09	.72	.02–.16	2.45	.015	.12
Gemma	.14	.73	.07–.21	3.87	<.001	.19
Qwen	.22	.73	.14–.29	5.93	<.001	.30
Llama	.15	.75	.07–.22	3.92	<.001	.20
**Ensemble**	.**15**	.**64**	**.08**–**.21**	**4**.**60**	**<.001**	.**23**

Mean, Average absolute discrepancy across all ratings in the testing sample; SD, standard deviation of absolute discrepancy ratings across all ratings in the testing sample; 95% CI, 95% confidence interval based on the standard error of measurement SEM for all signed discrepancies; OS *t*, One-Sample *t*-value; *d*, Cohen's d effect size.

### 3.3 Interrater reliability among artificial intelligence raters within each ensemble

To assess the extent to which fine-tuning procedures led our five different adaptable AI models to rate narratives in a similar manner, we calculated an additional set of ICCs that only used the ratings produced by our five AI raters. It is crucial to understand that these estimates do not provide an index of accuracy or validity, as they are calculated *without* consideration of the criterion ratings from the human experts. Instead, these ICCs describe the degree of agreement among AI raters. We report these to illustrate how fine-tuning AI raters can lead to similar ratings from very different AIs. For AFF, ICC estimates were notably high, resulting in a single-rater ICC (2, 1) of.89 and an average-rater ICC (2,5) of.98. For EIR, ICC estimates were also strong, with a single rater ICC (2, 1) of.88 and an average-rater ICC (2,5) of.97. In short, the five AI raters for each dimension showed very high levels of agreement with one another.

### 3.4 Comparing model performance prior to and after fine-tuning

Table 5 shows ICCs for AI raters prior to fine-tuning. Neither single-rater nor average-rater ICCs were near acceptable ranges for the EIR ensemble, and both were substantially lower than ICCs for AI raters following fine-tuning reported in Table 2. Though the AFF ensemble ratings are somewhat impressive, achieving a single-rater ICC of.69 (Moderate range) with the criterion ratings provided by expert humans and an average-rater ICC of.82 (Good Range), they are outshined by the post training single-rater and average rater ICCs which are both >.90 and in the excellent range. To more directly compare ratings prior to and following fine-tuning, we ran a series of paired *t*-tests using average absolute discrepancy with criterion ratings as the dependent variable. Findings are reported in Table 6. For AFF, mean absolute discrepancy decreased significantly from pre- to post-fine-tuning for all five AI raters and for the ensemble, with effect sizes indicating that differences were moderate to large (Cohen's *d* = 0.54–0.95). Similarly, mean absolute discrepancy for EIR decreased significantly for all raters and the ensemble following fine-tuning, with effect sizes ranging from moderate to large (*d* = 0.54–0.95).

**Table 5:** Single-Rater and average-rater intraclass correlation coefficients and 95% confidence intervals AI raters prior to fine-tuning .

	Single-Rater Intraclass Correlation Coefficients (ICC, 2,1)				Average-Rater Intraclass correlation coefficients (ICCs, 2,2)			
AI Rater	AFF ICC	95% CI	EIR ICC	95% CI	AFF ICC	95% CI	EIR ICC	95% CI
OpenChat	.73	.68–.78	.25	.15–.34	.85	.81–.88	.40	.27–.50
Mistral	.21	.11–.30	.29	.19–.38	.34	.19–.47	.45	.32–.55
Gemma	.60	.39–.72	.20	.06–.33	.75	.56–.84	.34	.12–.49
Qwen	.64	.58–.69	.34	.25–.42	.78	.73–.82	.51	.40–.59
Llama	.26	.01–.46	.15	.01–.28	.42	.01–.63	.26	.02–.44
**Ensemble**	.**69**	**.62**–**.75**	.**33**	**.21**–**.43**	.**82**	**.76**–**.86**	.**49**	**.35**–**.60**

AFF, Affective Quality of Representations Dimension; EIR, Emotional Investment in Relationships Dimension; ICC, Intraclass Correlation.

**Table 6:** Paired-Samples *t*-tests comparing absolute discrepancy prior to and after fine-tuning.

AI Rater	Pre M (SD)	Post M (SD)	Mean Diff [95% CI]	*t* (df)	*p*	*d*
AFF						
OpenChat	0.50 (0.60)	0.27 (0.43)	0.23 [0.18, 0.29]	8.19 (393)	<.001	0.54
Mistral	1.36 (1.25)	0.29 (0.44)	1.08 [0.95, 1.21]	16.19 (393)	<.001	0.82
Gemma	0.77 (0.72)	0.32 (0.47)	0.45 [0.37, 0.53]	11.31 (393)	<.001	0.57
Qwen	0.83 (0.74)	0.34 (0.48)	0.49 [0.40, 0.58]	11.16 (393)	<.001	0.56
Llama	1.49 (1.11)	0.37 (0.50)	1.13 [1.01, 1.24]	18.88 (393)	<.001	0.95
**Ensemble**	**0.66** (**0.49)**	**0.29** (**0.37)**	**0.37 [0.32, 0.43]**	**13.23** (**393)**	**<**.**001**	**0**.**67**
EIR						
OpenChat	1.04 (0.95)	0.48 (0.53)	0.56 [0.45, 0.66]	10.70 (398)	<.001	0.54
Mistral	1.34 (1.17)	0.47 (0.55)	0.87 [0.75, 0.99]	14.16 (398)	<.001	0.71
Gemma	1.49 (1.21)	0.48 (0.57)	1.01 [0.88, 1.14]	15.31 (398)	<.001	0.77
Qwen	1.10 (1.03)	0.50 (0.57)	0.60 [0.50, 0.70]	11.44 (398)	<.001	0.57
Llama	1.67 (1.32)	0.51 (0.58)	1.16 [1.03, 1.30]	16.44 (398)	<.001	0.82
**Ensemble**	**1.09** (**0.90)**	**0.15** (**0.64)**	**0.94 [0.83, 1.05]**	**16.61** (**398)**	**<**.**001**	**0**.**83**

AFF = Affective Quality of Representations Dimension; EIR = Emotional Investment in Relationships Dimension; Pre M (SD) = mean (standard deviation) of absolute discrepancy between AI rater and gold-standard expert rating prior to fine-tuning; Post M (SD) = mean (standard deviation) of absolute discrepancy following fine-tuning; Mean Diff. = mean reduction in absolute discrepancy from pre- to post-fine-tuning (Pre − Post), with 95% confidence interval; t (df) = paired-samples t statistic and degrees of freedom; d = Cohen's d effect size for the paired mean difference. All comparisons were statistically significant at *p* < .001.

## 4 Discussion

Our study explored whether adaptable AI models could be fine-tuned to serve as AI raters capable of assessing the affective-relational aspects embedded within narratives. Overall, fine-tuning was effective at producing AI raters able to assign ratings in a manner aligned with expert human psychologists. Present findings are similar to prior studies that have used prompt-engineering focusing on large, publicly available LLMs (48, 49). While the adaptable models used in the present study have far fewer parameters, they performed as well as their much larger counterparts. Given the level of rule complexity involved in narrative rating systems like the SCORS-G, the level of inter-reliability achieved is impressive. This level of proficiency would typically require a human to complete intensive, long-term training, possess domain expertise in clinical psychology, and have several years of practice. Comparing model performance prior to and after fine-tuning suggested that the training process drove gains in AI rater proficiency. This was most obvious for EIR, where models performed quite poorly prior to fine-tuning. For AFF, initial ICCs for a few of the models and the AFF AI ensemble overall were fairly impressive, possibly reflecting some similarities between the AFF scale and more general sentiment analysis for emotional content. Still, overall performance was significantly improved by the training process. While further validation and refinement of these AI raters may be necessary prior to deployment and adoption by health providers, findings suggest that they may be ready for use in human-in-the-loop approaches. Below, we discuss implications regarding AI raters for complex personality assessments, use of our AI raters for clinical and research aims, note limitations of this study, and suggest steps for future research.

The study joins with prior work in this area in suggesting that the linguistic capacities and inference abilities of adaptable LLMs, in the 3–7 billion parameter range, are sufficient to apply complex rating rules following training. While EIR and AFF may be relatively more straightforward relative to some other SCORS-G scales, they still involve the integration of multiple rules to make decisions across seven different rating levels (e.g., differentiating an EIR of 2 from an EIR of 3 requires integration of multiple rating principles). Nonetheless, fine-tuning raised performance to the level of human experts. To put the level of agreement achieved by our AI ensembles in perspective comparison with human experts is helpful. Across 13 studies using *highly experienced* expert human raters, average-rater ICCs for AFF ranged from.81 to.96 with a mean of.89 (*SD* = .04) and average-rater ICCs for EIR ranged from.78 to.97, with a mean of.90 (*SD* = .05) (70). This aligns with some recently conducted work using an incredibly complex, multi-rule system for rating psychological defenses (50) and with findings that AI's raters can validly assess complex features of personality (e.g., traits) from narratives (44). Prior studies have also found that, following PEFT, AI raters can effectively rate clinically-relevant constructs, such as alliance or provider adherence to treatment orientations (71, 72). Thus, while not the first to demonstrate this impressive capacity, our work adds to a growing body of research highlighting the utility of fine-tuning “lightweight” models to perform complex assessment tasks in health settings.

Consistent with prior work (48, 49, 53), use of an ensemble approach was superior to reliance on a single AI rater. While individual AI raters performed admirably, the ensemble score, the average of the ratings from five AI raters, yielded the highest levels of reliability and the lowest error rates. Aggregating judgments from distinct models may mitigate against idiosyncratic model-specific biases and stochastic variability, leading to a more robust assessment. Reliability estimates for ensemble ratings were strong, evidencing high agreement with ratings from human experts, who underwent rigorous training and have years of practice.

What might the path forward be for these specific AI raters? Our findings suggest two implementation pathways for the EIR and AFF raters: a human-in-the-loop approach and an automation approach. When reliability was assessed using average-rater ICCs, which assumes future ratings will be based on the average of a human expert (Rater 1) and an AI ensemble score (Rater 2), reliability estimates were notably strong. Thus, we currently advise this approach for employing AI raters in clinical settings. This would require a human to rate all patient narratives first and then the AI rating the narratives second. Ratings would then be compared and potentially averaged to produce final scores. Keeping a human in the loop leverages human domain expertise that can act as a check on AI performance. This process *also introduces AI into the loop*. AI acts as a second rater, providing a helpful check for the human rater. For example, clinicians could flag any narratives with absolute discrepancies between the human rater and AI ensemble of two or more for further analysis. Latter analysis could clarify if the AI ensemble score was inaccurate or if the human made an error. While rare, human error can occur. When it does, it can be due to issues like rater drift or fatigue. When human error is detected, it will be instructive for the rater. When the AI ensemble rating is off, this information could be fed back to this paper's authors and leveraged to further improve the AI raters going forward. This approach also moves clinical practice towards a best practice. In all settings, use of *multiple* raters for assessments is considered a best practice with psychometric benefits. Obviously, in most clinical settings having multiple human raters provide ratings is often not possible (for logistical reasons and sometimes for privacy reasons). By using an AI ensemble as a second rater, clinicians reap the psychometric benefits produced by multiple raters and key practical benefits of a second rater (e.g., flagging narratives that produce a discrepancy of >1 between the two raters for further analysis).

An important practical implication related to the use of these AI raters in clinical settings as part of a human-in-the-loop approach concerns some technical considerations. While fine-tuning the five AI raters required substantial computer power, the size of these models makes them comparatively lightweight. All five AI rater models used in this study fall within the ∼3-to-7 billion parameter range. These are small enough to be downloaded and run on a consumer-grade GPU or, with quantization, a standard laptop CPU. The current AI raters can be downloaded and run locally without modification. This matters for clinical and research settings where confidentiality is paramount. In many cases, sending psychotherapy narratives, autobiographical memories, therapy transcripts, or other sensitive material to third-party cloud-based APIs will raise data privacy concerns related to clinical ethics, IRB requirements, or data protection laws. While it still requires informed consent, local deployment may avoid this problem as data never leaves the user's own machine or network. This may make small, fine-tuned, open-weight models like these particularly well suited to health applications.

The second implementation approach is to use AI raters to automate the rating process. While desirable, this practice should be limited to research contexts at this time. Additional validation is necessary before AI raters can be used as the sole rater in clinical settings. There are many reasons for this. For example, there are differences in psychometric requirements of measurement tools used in clinical and research settings. When using any type of measure or assessment approach for research that employs a sample composed of several individuals, nonsystematic measurement error is likely to be randomly distributed across the sample. This minimizes the impact of this type of error on the study's findings and implications. However, when using assessment tools to assess a single individual, which is the case in clinical contexts, measurement error is not distributed across people. Thus, psychometric requirements (e.g., internal consistency; test-retest reliability) for clinical tools are more rigorous. Further, such tools require substantial validation and psychometric assessment prior to use. Even when they generate high agreement and good reliability metrics, raters may have biases or struggle to rate specific issues. These should be more rigorously examined prior to relying on our models as autonomous expert raters who do not need human oversight. Thus, additional research and validation of our AI raters must occur prior to using them to automate clinical ratings. By contrast, the level of agreement achieved here suggests that the current AI raters, even if imperfect, are likely to be useful for research focusing on large groups of individuals. When employed for this purpose, using our AI raters to fully automate ratings is an option at this time. When doing so, this should obviously be acknowledged by the researcher, and the average-rater ICC should be reported to indicate level of agreement with the AI raters within the ensemble.

The capacity to automate ratings in research settings is important, as many challenges associated with narrative rating systems are most notable in research contexts. Rating seven to twelve narratives collected in a clinical protocol can be mildly more burdensome than scoring a brief self-report measure, but it pales in comparison to rating several hundreds to thousands of narratives produced by a single study. Thus, we anticipate that AI raters will produce the most positive impacts for clinical researchers at this time. This too may yield clinical benefits, even if they are indirect. For example, to clarify the clinical strengths and limitations of narrative assessment systems, something useful to clinicians, the pace and scale of narrative research *must* increase. Ironically, research in this domain has slowed since the 1980s, both relative to its own earlier pace and relative to research in other areas of psychology. It is unlikely this shift reflects a lack of interest, given that the benefits of narrative assessments, as part of a multi-method assessment approach, are widely respected (14–18, 28–41). Instead, this shift is likely to reflect practical challenges associated with this work. Narrative research is demanding: it relies on human raters, who require months to years of training to achieve expert levels of proficiency and considerable time to rate the narratives. Researchers may deal with such challenges by limiting sample sizes, but this is not an optimal solution. Most narrative assessment studies make use of small samples compared to self-report studies. Larger narrative studies do exist, but they require a substantial amount of time. Collectively, these challenges act as a barrier to entry for clinical researchers who would otherwise be likely to employ these methods. To put this in context, the first author conducts narrative-based (e.g., narrative assessment; psychotherapy process research) *and* self-report research. His narrative-based studies require several months *following* data collection just to finalize narrative ratings. By contrast, scale scores for self-report studies can easily generated within 48 h of the end of data collection. As such, his self-report studies are more expedient, as he can rapidly move from data collection to analysis to writing manuscripts. In the publish-or-perish world of academia, it is easy to see how this discrepancy could incentive researchers to gravitate towards relying more and more on self-report methods. To be clear, we are not against self-report measures, which are incredibly useful tools. Our concerns lie with *sole* reliance on this method, as best practices call for multi-method approaches in research and clinical settings (13, 15).

For multi-method approaches to be embraced, clinicians and researchers must view them as logistically feasible. Automating narrative ratings would speed up narrative research, resulting in a pace that may approach that of self-report studies, effectively lowering the bar for researcher entry, and potential to increase the size and pace of narrative research. All of these allow the field to more rapidly explore and answer key questions (e.g., How do self-report and narrative-based methods intersect and diverge? What are the strengths of narrative-based measures and the areas of weakness?).

Despite promising findings, it is important to consider the limitations associated with this study. The narratives used to craft our AI raters were drawn from training materials and existing research samples rather than from live, real-world clinical cases. Prior to deployment for use in clinical settings, it is essential to validate them using data samples containing narratives derived from clinical populations in order to ensure ecological validity. Further, the vast majority of narratives in this study were stories generated in response to picture-story tasks. While the TAT and picture-story tasks more generally are commonly used in personality assessments, it is unclear if our raters would provide accurate ratings for other types of narrative data the SCORS-G has been used to rate (e.g., Therapy Sessions; Autobiographical Memories; Relational Episodes). Further validation of our AI raters with other types of narratives is necessary to establish if these models generalize to other types of narrative content. A similar limitation concerns our samples. The majority of narratives were from Samples B and C, both of which consisted predominantly of young adult, college-age populations from the United States. Whether AI rater performance generalizes to non-Western, older, or clinical populations remains untested. While our approach is demographic-agnostic in principle, which may make performance more portable across groups, this remains to be empirically confirmed. Because our AI raters are lightweight enough to run locally rather than requiring proprietary API access, researchers working with more diverse samples, including those with limited computing budgets, can directly test these models' generalizability within their own populations. Relatedly, identifying partners with more diverse samples, or datasets containing additional narrative types (e.g., autobiographical memories), would offer a valuable avenue for jointly assessing generalizability across both populations and narrative types. This study also used ratings from expert-based human raters as criterion ratings. This treats these ratings as similar to an objective “ground truth.” While imperfect, these are still useful for determining if AI can perform at a human experts currently making assessments. This study also focused exclusively on the affective-relational dimensions of the SCORS-G. While these are among the most strongly and consistently associated with health outcomes and risk in mental health settings, the system contains other dimensions to assess cognitive aspects of representations and aspects of self-experience, such as self-esteem. Additional research is needed to establish that the process used in this paper can be effective in developing AI raters for the other SCORS-G dimensions assessing self-functioning and social-cognitive features of personality. This would result in a more comprehensive set of assessment tools.

There are also some limitations associated with the technical aspects of the study. We employed AI models that contain ∼3-to-7 billion parameters. These proved effective and efficient, and we selected them because they are available to health researchers and logistically more feasible to use relative to larger models; however, it remains an open question whether larger models would yield greater accuracy or if smaller, more specialized models can achieve similar performance with even greater efficiency. Future studies using similar methods to this study could generate models using larger or smaller adaptable LLMs and compare findings with those from the present study. Relatedly, although our fine-tuning protocol incorporated safeguards against overfitting (e.g., low-rank parameter updates, dropout, a short, fixed training schedule), we did not monitor validation loss during training or implement early stopping. As a result, we cannot rule out the possibility that model performance could have been further improved had training been guided by a validation-based stopping criterion rather than a fixed epoch count. Additionally, we used 4-bit NF4 quantization to make fine-tuning of 7-billion-parameter models feasible on a single GPU; while prior work indicates QLoRA closely approximates full-precision fine-tuning performance, we did not conduct a full-precision ablation to confirm this directly for our models and tasks, and future work should verify this empirically. While not necessarily limitations, two final points are noteworthy. The first concerns efforts to understand “how” AI raters are assigning ratings. Even when fine-tuned models, such as the ones we developed, achieve good reliability with human experts, they effectively function as “black boxes,” producing numeric outputs without explaining their ratings. We made no attempt to have our AI raters focus on thematic narrative content (note, AI raters can be instructed to provide brief explanations for ratings, but these were not investigated as part of this study). Two complementary approaches may be promising for future researchers to study “how” AI raters are providing their ratings. First, chain-of-thought prompting or evidence-citation fine-tuning could enable the model to note the specific narrative features (e.g., words, phrases, or themes) that drive a rating. These could potentially be studied to identify themes, patterns, and even possible errors in logic or application. A second approach involves the use of *post-hoc* attribution methods, such as Shapley Additive Explanations (SHAP) or Local Interpretable Model-Agnostic Explanations (LIME), that seek to identify which aspects of narratives influence each AI raters' scoring. Both approaches would enhance interpretability and deepen our theoretical understanding of how AI raters ‘read’ narratives.

The second point relates to the prior one but differs in emphasis. In psychological assessment contexts, systems like the SCORS-G provide frameworks for quantifying prespecified aspects of narratives, such as affective tone and quality and depth of relational experiences; Clinicians, however, may also leverage thematic content from individual narratives to generate person-specific clinical hypotheses that go beyond the system's prespecified framework. Thus, clinicians' thematic content analysis serves to complement quantitative frameworks, like the SCORS-G. It is possible that AI raters could be developed to engage in thematic content analyses. We did not explore this within our study, as it was beyond our scope. Nonetheless, this could be a worthy focus for future investigators. For example, if LLM raters can apply complex, rule-governed rating systems to narrative content, they could likely be adapted to perform qualitative tasks such as identifying recurrent themes across a set of narratives from a single respondent or for flagging narrative features that co-occur with particularly high or low scores on scales like AFF and EIR. While beyond the scope of the present study, a line of research focusing on thematic evaluation thematic assessment could be useful and even help inform how AI raters assign ratings @@ (73).

## Data Availability

The data analyzed in this study is subject to the following licenses/restrictions: Data Ownership. Requests to access these datasets should be directed to csiefert@umich.edu.
