# Supplementary material for: From free association to free parameters: machine-learning empowers artificial intelligence models to rate the affective-relational aspects of narratives like an expert psychologist
Source: Front Digit Health. 2026 Aug 27;8:1762253. doi: 10.3389/fdgth.2026.1762253 (PMC13558173; doi:10.3389/fdgth.2026.1762253)
Supplement: Supplementary file 1 [file Datasheet1.docx]

Supplemental Material 1: Pre-Pended Prompts

Prepended Prompt for Emotional Investment in Relationships (EIR).

*You rate narratives on a 1 to 7 scale for Emotional Investment in Relationships using these rules. 1 = Violent, abusive, harmful, or neglectful interpersonal interactions. Interactions are much more negative than positive. 2 = Negative but not abusive interactions (e.g., betrayal, conflict, lasting mistrust; theft). Rate 2 if the character intends violence but is prevented from acting on it. Rate a 2 if interactions produce more negative interpersonal impacts than positive. Rate 2 if only one person is present and there are no real or imagined interpersonal interactions. 3 = Minimal or superficial interactions (e.g., interaction with store clerk; briefly talking to a stranger) or mildly negative interactions (e.g., peer pressure; disappointment with others; mild arguments; mild dishonesty; regretful mourning; being criticized). Rate a 3 if interactions produce slightly more negative interpersonal impacts than positive. 4 = Neutral/professional or mixed interaction (roughly the same amount of negative and positive interpersonal impact). 5 = Positive personal interactions involving warmth, care, or support or interactions produce more positive interpersonal impacts than negative. 6 = Strong emotional investment, relationship depth, clear intentions to be kind, helpful, supportive, loving; and warm. Interactions produce positive interpersonal impacts without any negative impacts. 7 = Positive interpersonal interactions that suggest deep connection, long-lasting impact; mutual support; transformative relationships. Descriptions of interactions are detailed. Interactions produce notably positive impacts and there are no negative impacts. Output Format = "Rating = <number between 1 - 7>*

Prepended Prompt for Affective Quality of Representation (AFF)

*You rate narratives on a 1 to 7 scale for the Affective Quality of Representations (AFF) using these rules. 1 = a character commits a malevolent, abusive, or physically harmful acts, including extreme verbal abuse, suicide, rape, murder, or physical/sexual abuse, during the story. The harmful interaction must occur during the story. 2 = story contains intense negative emotions without any positive emotions or positive relational interaction or story has no positive emotions and has themes of intense interpersonal rejection, hostility, neglect, or non-violent crime. 3 = narrative primarily contains mild-to-moderate negative emotions, more negative than positive emotion, characters have highly negative self-evaluations, or story has negative and positive emotions but ends in a way that emphasizes negative emotion. 4 = emotional descriptions are bland, limited, absent or emotions are neutral or the amount of positive and negative emotions is so balanced that it is unclear if the emotional tone is more negative or positive. 5 = narrative includes either mild positive emotions, includes more positive than negative emotions, or a story is moderately negative, but ends in a positive or uplifting way. 6 = there is a moderate amount of positive emotion and no negative emotions are present or there are no negative emotions and interpersonal interactions are depicted in a clearly positive manner (e.g., compassion; emotional support; close connection, trust, and shared positive feelings). 7 = narrative is extremely positive, relationships are depicted in notably positive ways, and there are no negative emotions. To score 7, positive emotions and relational interactions must be described in considerable detail and exhibit intense joy, fulfillment, or deep connection.*

*Output Format = "Rating = <number between 1 - 7>*

Narrative:

"Mathew was having a terrible day at work. His boss had yelled at him over something small, there was bad traffic, he really just could not wait to get home. However, when he arrives home, he finds that his wife had been drinking again., and she was passed out naked on the first floor bathroom. He was angry with her, but knew it was no use scolding her in this state He carries her upstairs and tucks her in bed. At that moment, all of that day’s hardships rush through him and he starts crying in the middle of the room. After about an hour, he picks himself up into the bathroom and takes a long and hot shower while he silently cries some more. He then falls asleep and tries his best to keep his spirits high the next day. (141)"

<assistant>: Rating = 3

Explanation:

- **Identify and Evaluate Emotion Words**: The narrative contains a mix of negative emotions such as anger, frustration, sadness, and crying. While there is one positive action (carrying his wife to bed), it does not seem to bring immediate relief or comfort. The overall emotional tone leans more towards the negative.

- **Evaluate Emotional Aspects of Relationships/Interpersonal Interactions**: The relationship between Mathew and his wife seems strained due to the wife's drinking issues. There is no clear indication of emotional support or positive relational interactions. The interaction ends with Mathew silently crying, which suggests a lack of resolution or comfort.

- **Consider Story Structure**: The narrative ends with Mathew trying to keep his spirits high the next day, but given the context, it's not clear if this will lead to a positive outcome. The emotional tone of the ending is uncertain and somewhat unresolved.

- **Assign a Rating**: Given the mix of negative emotions and the absence of clear positive emotional outcomes, the rating is 3. The narrative primarily contains mild-to-moderate negative emotions or more negative than positive emotion, with little to no positive relational interactions.
