# Supplementary material for: From free association to free parameters: machine-learning empowers artificial intelligence models to rate the affective-relational aspects of narratives like an expert psychologist
Source: Front Digit Health. 2026 Aug 27;8:1762253. doi: 10.3389/fdgth.2026.1762253 (PMC13558173; doi:10.3389/fdgth.2026.1762253)
Supplement: Supplementary file 2 [file Datasheet2.docx]

**Supplemental Material 2**

*Pre-Fine-Tuning Baseline Model Performance*

**Affect and Investment in Relationships (AFF)**

Table 1

*Distribution of Absolute Discrepancies and Percentage of Ratings Within Absolute Discrepancy Ranges for Pre-Fine-Tuning AFF Ratings (N = 394)*

| **AI Rater** | **Mean** | **SD** | **95% CI** | **Total % Within Limits** | **No Discrepancy** | **Within Tolerance** | **Mild Deviation** | **Moderate Deviation** | **Critical Error** |
| --- | --- | --- | --- | --- | --- | --- | --- | --- | --- |
| OpenChat | .50 | .60 | .45 – .56 | 91% | 49% | 41% | 5% | 0% | 4% |
| Mistral | 1.36 | 1.25 | 1.24 – 1.49 | 55% | 27% | 28% | 6% | 0% | 39% |
| Gemma | .77 | .72 | .70 – .84 | 79% | 35% | 44% | 7% | < 1% | 14% |
| Qwen | .83 | .74 | .76 – .90 | 83% | 28% | 54% | 3% | < 1% | 14% |
| Llama | 1.49 | 1.11 | 1.38 – 1.60 | 49% | 18% | 31% | 10% | < 1% | 41% |
| **Ensemble** | **.66** | **.49** | **.61 – .71** | **80%** | **6%** | **75%** | **14%** | **5%** | **2%** |

Note. Mean = Average absolute discrepancy across all ratings in the testing sample; SD = standard deviation; 95% CI = 95% confidence interval based on the standard error of measurement; Total % Within Limits = percentage of ratings with an absolute deviation from zero to one; No Discrepancy = percentage with absolute discrepancy of zero; Within Tolerance = percentage with absolute discrepancy between .01 and one; Mild Deviation = percentage with absolute discrepancy of 1.01 to 1.50; Moderate Deviation = percentage with absolute discrepancy of 1.51 to 1.99; Critical Error = percentage with absolute discrepancy of two or more.

Table 2

*Descriptive Statistics and Tests of Mean Signed Discrepancy by Rater for Pre-Fine-Tuning AFF Ratings (N = 394)*

| **AI Rater** | **Mean** | **SD** | **95% CI** | **OS t** | **p** | **d** |
| --- | --- | --- | --- | --- | --- | --- |
| OpenChat | 1.07 | 1.44 | .92 – 1.21 | 14.66 | < .001 | .74 |
| Mistral | 1.11 | 1.81 | .94 – 1.29 | 12.26 | < .001 | .62 |
| Gemma | -.50 | .93 | -.59 – -.41 | -10.69 | < .001 | -.54 |
| Qwen | -.55 | 1.84 | -.73 – -.36 | -5.90 | < .001 | -.30 |
| Llama | -4.59 | 1.43 | -4.73 – -4.45 | -63.71 | < .001 | -3.21 |
| **Ensemble** | **-.10** | **.73** | **-.17 – -.03** | **-2.66** | **.008** | **-.13** |

Note. Mean = Average signed discrepancy across all ratings in the testing sample; SD = standard deviation; 95% CI = 95% confidence interval based on the standard error of measurement; OS t = One-Sample t-value; d = Cohen's d effect size.

**Emotional Investment in Relationships (EIR)**

Table 3

*Distribution of Absolute Discrepancies and Percentage of Ratings Within Absolute Discrepancy Ranges for Pre-Fine-Tuning EIR Ratings (N = 399)*

| **AI Rater** | **Mean** | **SD** | **95% CI** | **Total % Within Limits** | **No Discrepancy** | **Within Tolerance** | **Mild Deviation** | **Moderate Deviation** | **Critical Error** |
| --- | --- | --- | --- | --- | --- | --- | --- | --- | --- |
| OpenChat | 1.04 | .95 | .95 – 1.13 | 69% | 29% | 40% | 9% | < 1% | 22% |
| Mistral | 1.34 | 1.17 | 1.22 – 1.45 | 61% | 21% | 40% | 8% | 0% | 32% |
| Gemma | 1.49 | 1.21 | 1.37 – 1.61 | 53% | 20% | 33% | 8% | 0% | 39% |
| Qwen | 1.10 | 1.03 | 1.00 – 1.20 | 69% | 27% | 43% | 7% | 0% | 24% |
| Llama | 1.67 | 1.32 | 1.54 – 1.80 | 50% | 17% | 34% | 7% | 0% | 42% |
| **Ensemble** | **1.09** | **.90** | **1.00 – 1.18** | **57%** | **7%** | **50%** | **16%** | **10%** | **17%** |

Note. Mean = Average absolute discrepancy across all ratings in the testing sample; SD = standard deviation; 95% CI = 95% confidence interval based on the standard error of measurement; Total % Within Limits = percentage of ratings with an absolute deviation from zero to one; No Discrepancy = percentage with absolute discrepancy of zero; Within Tolerance = percentage with absolute discrepancy between .01 and one; Mild Deviation = percentage with absolute discrepancy of 1.01 to 1.50; Moderate Deviation = percentage with absolute discrepancy of 1.51 to 1.99; Critical Error = percentage with absolute discrepancy of two or more.

Table 4

*Descriptive Statistics and Tests of Mean Signed Discrepancy by Rater for Pre-Fine-Tuning EIR Ratings (N = 399)*

| **AI Rater** | **Mean** | **SD** | **95% CI** | **OS t** | **p** | **d** |
| --- | --- | --- | --- | --- | --- | --- |
| OpenChat | .01 | 1.41 | -.13 – .15 | .11 | .916 | .01 |
| Mistral | -.44 | 1.72 | -.61 – -.27 | -5.08 | < .001 | -.25 |
| Gemma | -.95 | 1.67 | -1.12 – -.79 | -11.42 | < .001 | -.57 |
| Qwen | .14 | 1.50 | .00 – .29 | 1.90 | .058 | .10 |
| Llama | -1.24 | 1.73 | -1.41 – -1.07 | -14.32 | < .001 | -.72 |
| **Ensemble** | **-.50** | **1.32** | **-.63 – -.37** | **-7.51** | **< .001** | **-.38** |

Note. Mean = Average signed discrepancy across all ratings in the testing sample; SD = standard deviation; 95% CI = 95% confidence interval based on the standard error of measurement; OS t = One-Sample t-value; d = Cohen's d effect size.
